# Characterization of *Pseudomonas aeruginosa* Phage C11 and Identification of Host Genes Required for Virion Maturation

**DOI:** 10.1038/srep39130

**Published:** 2016-12-21

**Authors:** Xiaoli Cui, Jiajia You, Li Sun, Xiaojing Yang, Tian Zhang, Kechong Huang, Xuewei Pan, Fenjiao Zhang, Yang He, Hongjiang Yang

**Affiliations:** 1 Key Laboratory of Industrial Microbiology, Ministry of Education, Tianjin Key Laboratory of Industrial Microbiology, College of Biotechnology, Tianjin University of Science and Technology, Tianjin 300457, China

## Abstract

The underlying mechanisms of phage-host interactions largely remained to be elucidated. In this work, *Pseudomonas aeruginosa* phage C11 was first characterized as a *Myoviridae* virus having a linear dsDNA molecule of 94109 bp with 1173 bp identical terminal direct repeats (TDR). Then the mutants resistant to phage C11 were screened in a Tn5G transposon mutant library of *P. aeruginosa* PAK, including two mutants with decreased adsorption rates (DAR) and five mutants with wild-type adsorption rates (WAR). When the WAR mutants were incubated with phage C11, their growth rates were significantly inhibited; the replication of the phage genomic DNA was detected in all the WAR mutants with the real-time quantitative PCR analysis; and the synthesized phage genomic DNA was processed into monomers for packaging evidenced by the southern blot analysis. Moreover, with strain PAK as indicator, small quantities of phage C11 were synthesized in the WAR mutants. Taken together, these data suggested the identified genes of the WAR mutants are necessary for efficient synthesis of the infectious phage particles. Finally, the WAR mutants were detected sensitive to two other *Pseudomonas* phages closely related with C11, further implying the evolved diversity and complexity of the phage-host interactions in both sides.

Phage therapy shows great promises in combating bacterial infections^1^,^2^. Candidate phages used for treatments are usually selected mainly based on their host ranges. Phages with broader host ranges will likely target more bacterial strains and may have more potential applications in practice. In addition to killing spectrums of phages, antibacterial efficacy of phages is another key aspect of candidate phages for consideration of phage therapy, which is involved a variety of genes from both sides in host-phage interactions^3^,^4^. A number of mechanisms have been found contributing the defense of phage attacks in many bacteria^5^,^6^. All these involved pathways are employed by diverse bacteria strains in the active strategies against phage infections. The host genes necessary for phage proliferations largely remain to be identified and elucidated.

The systematic investigations of phage-host interactions between *Escherichia coli* and the relevent phages have been performed recently. Two bacterial libraries, the Keio collection and the ASKA library, have been used for the genome-wide searches of host genes involved in the phage T7 infection, including some genes for their ability to inhibit growth of T7 phage and the other genes required for the virus infection^7^. The Keio collection was also used to identify bacterial genes involved in the λ phage infection process. Totally 57 host genes were identified, the majority of them had not been found associated with phage infections previously^8^. Phage receptor related genes were also screened in *E. coli* phage mEp213 infection by employing a novel strategy to select bacterial cell-envelope mutants resistant to phage infection^9^. More recently, it’s found that the genome injection of *E. coli* phage HK97 required the glucose transporter PtsG and the periplasmic chaperone FkpA encoded by the host genes^10^.

Similar studies on host-phage interactions have also been carried out in various systems, including *Bacillus subtilis* and phage SPP1^11^, *Yersinia pestis* and phage L-413C^12^, *Vibrio cholerae* and phageVP3^13^, *V. cholerae* biotype El Tor and phage VP4^14^, and *Salmonella enterica* serovar Typhi and a number of diverse phages^15^,^16^,^17^. All identified host genes required for phage infections approximately fall into two groups, one group of the genes related to the receptors syntheses, involving in phage recognitions and adsorptions; the other group of the genes involved in various pathways, possibly functioning in the stages of phage infection other than the adsorption.

*Pseudomonas aeruginosa* is an opportunistic pathogen causing a number of diseases in human beings and also one of the most common bacteria causing nosocomial infections^18^. *P. aeruginosa* possesses a relatively large genome harboring multiple drug resistance determinants, leading to the ever increasing prevalence of multi-drug resistances in clinical isolates along with the imprudent and excessive use of antibiotics^19^,^20^. It’s urgent to identify antibiotic alternatives with efficacious antibacterial activities^21^. Phages are able to specifically kill bacteria hosts with high efficiency and expected to be used in bactericidal treatments as the biological agents^22^,^23^. To date, a few clinical trials have been carried out using phages against *P. aeruginosa* infections with the encouraging results, such as leg ulcers^24^, burns^25^, and ear infection^26^. Phage treatment has also been conducted to control *P. aeruginosa* infection in cystic fibrosis patients in a few cases^27^. New techniques have been applied in the exploration of host-phage interactions in a few systems, including *P. aeruginosa* and phage LUZ19^28^, *P. aeruginosa* and phage PaP3^29^, and *P. aeruginosa* and phage PAK_P3^30^. The data reveal the global changes in host cells when infecting with the virulent phages. A number of phage genes are found playing crucial roles during the infections. On the other hand, host genes necessary for phage infections have also been investigated in some *Pseudomonas* strains with classical genetic techniques. The majority of the identified genes are related to the synthesis of phage receptors, such as type IV pili and LPS^3^,^28^,^31^. In screening phage JG004 resistant mutants, the gene *speD* encoding S-adenosylmethionine decarboxylase proenzyme for polyamine biosynthesis was identified, and the enzyme is possibly involved in the phage genome packaging process by affecting the charge density of the genomic DNA molecules^32^.

Phage C11 is a virulent virus isolated previously using the clinical strain *P. aeruginosa* TJC422 as indicator^33^. In this work, the characteristics of phage C11 were evaluated. The genome of phage C11was sequenced, curated, and annotated. In the investigation of the host-phage interaction, the mutants resistant to phage C11 were screen in a Tn5G transposon insertional library of strain PAK and the interrupted host genes were identified. The mechanisms underlying the infection process was further explored by characterization of the isolated phage-resistant mutants.

## Results

### Biological characteristics of phage C11

**Figure 1:**
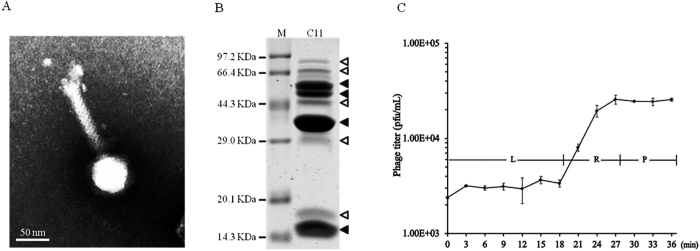
Characteristics of phage C11. (**A**) Transmission electron micrograph of phage C11. The bar represented a length of 50 nm. (**B**) SDS-PAGE analysis of the phage C11 particles. Solid triangles pointed at the major protein bands. Open triangles pointed at the minor protein bands. (**C**) One-step growth curve of phage C11. L represented the latent phase. R represented the rise phase. P represented the plateau phase. The experiment involved triplicate cultures.

Transmission electron micrograph (TEM) was used to investigate morphology of phage C11. The images showed that phage C11 had an icosahedral head of 65 nm in diameter and a long contractile tail with a length of 122 nm, indicating that phage C11 can be tentatively classified into the family *Myoviridae* (Fig. 1A). In the SDS-PAGE analysis of phage particles, 4 major bands and 5 minor bands were observed on the gel, and the protein bands likely represented the major structural proteins of phage C11 (Fig. 1B). Furthermore, the infectivity of phage C11 was characterized by one-step growth experiment at a multiplicity of infection (MOI) of 0.001. In the infection process of strain PAK, phage C11 had a latent period of about 18 min and a burst size of approximately 11 PFU/infection centre (Fig. 1C).

### Termini identification of the phage C11 genome

**Figure 2:**
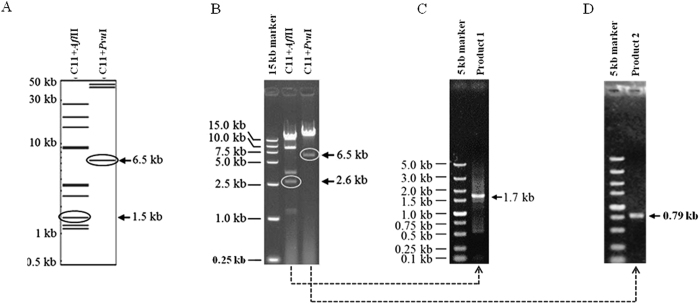
Identification of the genome termini of phage C11. (**A**) The restriction digestion simulation of the linear phage genomic DNA with the enzymes *Pvu*I and *Afl*II, respectively. (**B**) The electrophoresis pattern of the digested phage C11 genomic DNA. The 6.5 kb *Pvu*I fragment might include the 3′ terminus of phage C11 genome. The 2.6 kb *Afl*II fragment might include the 5′ terminus of phage C11 genome. (**C**) The 2.6 kb *Afl*II fragment was sequentially subjected to filling-in ends with klenow enzyme, ligation with T4 DNA ligase, and amplification using the primers 5 F and 5 R. The 1.7 kb amplicon (product 1) was sequenced with the primers 5 F and 5 R to determine the sequence of 5′ terminus. (**D**) The 6.5 kb *Pvu*I fragment was sequentially subjected to filling-in ends with klenow enzyme, ligation with T4 DNA ligase, and amplification using the primers 3 F and 3 R. The 0.79 kb amplicon (product 2) was sequenced with the primers 3 F and 3 R for the analysis of 3′ terminus.

The assembled phage genome was 93010 bp in length and it was subsequently confirmed as a linear dsDNA molecule by digestion analysis with the enzyme *Pvu*I (Fig. 2A,B). To further verify the phage genomic structure, the high-frequency sequence (HFS) sites possibly representing the phage genome termini were revealed by analyzing the sequencing depth^34^. One HFS between 82259 bp through 83429 bp of the assembled genome was detected and hypothesized as the genome termini sites. In combination with the restriction mapping analysis, the 6.5 kb *Pvu*I fragment and 2.6 kb *Afl*II fragment (larger than the simulated 1.5 kb fragment) possibly containing the phage termini were purified from gel, respectively. After amplification of the purified fragments, the 1.7 kb and 0.79 kb PCR products were sequenced (Fig. 2A–D). An identical terminal direct repeat sequence of 1173 bp was found in both the two fragments and the genome size of phage C11 was curated to 94109 bp.

### Genome annotation

**Figure 3:**
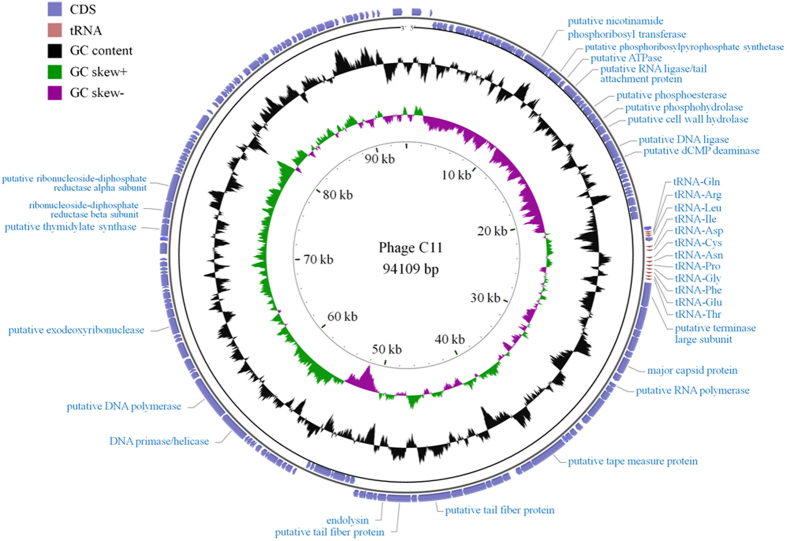
Analysis of the phage C11 genome. The outmost two rings showed CDS features in blue boxes on both the plus and minus strands. CDS with no annotations represented hypothetical proteins. The clustered transfer RNA genes were abbreviated as Gln (glutamine), Arg (arginine), Leu (leucine), Ile (isoleucine), Asp (aspartic acid), Cys (cysteine), Asn (asparagine), Pro (proline), Gly (glycine), Phe (phenylalanine), Glu (glutamate), and Thr (threonine). The black ring showed the GC content of the phage C11 genome. The inmost ring showed the value of GC skew, green color standing for positive and purple for negative.

A total of 172 ORFs encoding protein were predicted of the phage C11 genome, including 30 proteins with known putative functions and 142 hypothetical proteins (Table 1). The overall genome structure showed that genes with similar functions were arranged in clusters, which were divided into five functional modules: the module of nucleotide metabolism related genes; the module of the head and tail of phage and cell lysis related genes; the module of DNA replication, transcription, recombination, and modification related genes; and the remaining two modules having a number of genes localized close to the termini of the genome with unknown functions (Fig. 3). Between the modules of nucleotide metabolism related genes and cell lysis genes, 12 tRNA genes were predicted and closely colocalized within one cluster (Fig. 3). Phage C11 genome was highly homologous to phage JG004 (NC_019450), phage PaP1 (NC_019913), phage vB_PaeM_C2-10_Ab1 (NC_019918), and phage PAK_P1 (NC_015294) at the nucleotide level of 95.32%, 92.41%, 92.11%, and 93.03%, respectively, and with very similar genome structures. However, genes *ORF158, ORF159*, and *ORF165* were unique present in the phage C11 genome encoding the proteins with unknown functions.

### Isolation and characterization of phage resistant mutants

**Figure 4:**
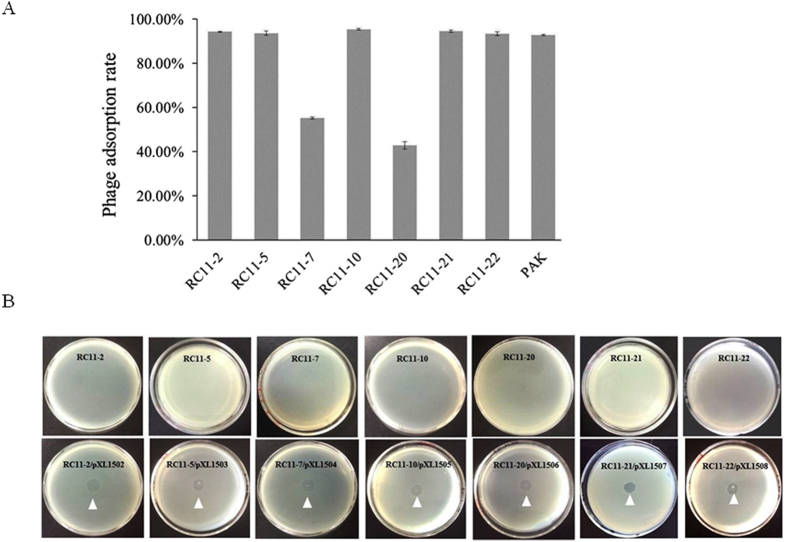
Characterization of the phage resistant mutants. (**A**) Phage adsorption rate of the mutants and the parent strain PAK. The experiment was repeated three times. (**B**) Complementation experiment. The upper panel showed the mutants were resistant to phage C11. The lower panel showed the sensitivity restoration to phage C11 in the corresponding transformants. White triangles pointed at the clear zones caused by spotting the 100-fold diluted phage C11 lysate (10^8^ pfu/ml) on the double-layer plates.

By screening the Tn5G transposon insertional library, seven mutants resistant to phage C11 were acquired from the collection of nearly 20,000 mutants. In the spotting assay, strain PAK displayed transparent zones within 4 h incubation; whilst no clear zones were observed in the lawns formed by the mutants. Additionally, no plaques were formed in the double-layer plating assay using the mutants as indicators. The data implied that the isolated mutants showed resistance to phage C11 infections. The adsorption rate of the mutants was determined, two phage-resistant mutants displaying decreased absorption rates (DAR) of 55.3% and 42.8%, respectively, significantly lower than that of the parent strain PAK (all P values less than 0.01); while the remaining five phage-resistant mutants displaying wild-type adsorption rates (WAR) at the range from 93.3% to 95.4%, no significant difference from the adsorption rate 92.7% of PAK (all P values higher than 0.05) (Fig. 4A).

### Identification of the disrupted genes

The Tn5G transposon insertion sites were determined by the inverse PCR method. The DAR mutants RC11-7 and RC11-20 had the decreased adsorption rates, suggesting the disrupted genes might be related with the phage receptor synthesis. The prolonged measurement of the adsorption rate further confirmed that both RC11-7 and RC11-20 had their adsorption ability impaired (Fig. S1). RC11-7 had the transposon inserted in gene *PAK_02041* identical to gene *wbpR* of *P. aeruginosa* LESB58, which encodes a glycosyltransferase involved in LPS synthesis^35^. RC11-20 had the transposon inserted in gene *PAK_05691* identical to gene *PA5181* of *P. aeruginosa* PAO1, which encodes a probable molybdopterin oxidoreductase and it’s the second gene of the operon *PA5180-PA5181* (www.pseudomonas.com).

The five WAR mutants all had the wild-type adsorption rates similar to strain PAK, indicating the disrupted genes were not related to the synthesis of the phage C11 receptor. RC11-2 had the transposon inserted in the intergenic region between gene *PAK_03116* and gene *PAK_03117* of PAK. The identical sequence is found and annotated as gene *BN889_05221* of *P. aeruginosa* PA38182, which encodes a hypothetical peptide of 75 aa without any known function. RC11-5 had the transposon inserted in the sequence identical to the intergenic region before gene *PA3808* of *P. aeruginosa* PAO1, which is the last gene in the operon *iscR-iscS-iscU-iscA-hscB-hscA-fdx2-PA3808*. The proteins encoded by the operon are mainly responsible for the iron-sulfur cluster (ISC) assembly. RC11-21 had the transposon inserted in gene *PAK_00453* identical to gene *PA0243* of *P. aeruginosa* PAO1, which encodes a TetR bacterial regulatory protein with helix-turn-helix (HTH) signature (PF00440). RC11-22 had the transposon inserted in gene *PAK_04254* identical to gene *PA1115* of *P. aeruginosa* PAO1, which encodes a probable sulfatase (PF00884) catalyzing the hydrolysis of sulfate esters. RC11-10 had the transposon inserted in gene *PAK_03341* identical to gene *PA1993* of *P. aeruginosa* PAO1, which encodes a probable major facilitator superfamily (MFS) transporter (www.pseudomonas.com).

### Complementation of the phage resistant mutants

To verify if the interrupted genes were responsible the phage-resistant phenotypes, the target genes were cloned into the vector pUCP18 with their natural promoters or driven by the *Plac* promoter carried by the plasmid (Table 2) and the recombinant plasmids were transformed into the mutants. The resulting transformants were analyzed for their sensitivity to phage C11. The analyses showed that the sensitivity to phage C11 was restored in all seven phage-resistant mutants by the trans-complementation of the corresponding target genes, demonstrating the disrupted genes were responsible for the phage-resistant phenotype of the mutants (Fig. 4B).

### Phage C11 affecting the growth dynamics of the WAR mutants

**Figure 5:**
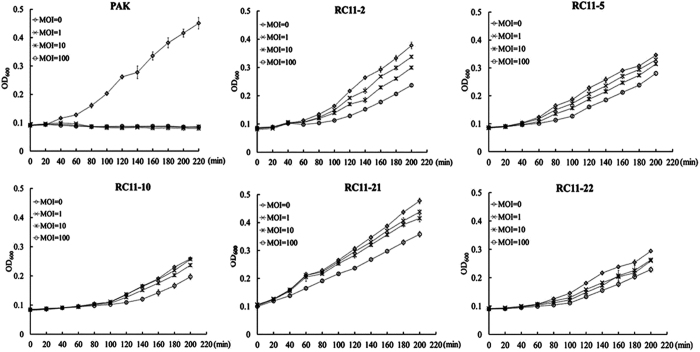
The inhibitory effect of phage C11 on the growth rates of the WAR mutants and the parent strain PAK. MOI: the multiplicity of infection.

The fact that the WAR mutants had the wild-type phage adsorption rates indicated that the impaired genes were not involved in the process of phage adsorption. These genes were possibly associated with the stages of the phage genome injection, replication, packaging, or progeny release. Based on this hypothesis, the WAR mutant cells were mixed with phage C11 at different MOIs and the growth rates were measured. As the MOIs increased from 1 to 100, the WAR mutants grew slower as the more phage particles added (Fig. 5). The data showed that the phage-resistant WAR mutants exhibited fitness costs when infected with phage C11. The injected phage genome may initiate a series of processes, such as transcription, translation, and replication, consuming extra building blocks and energy. Growth inhibitors encoded by the phage genes would be another reason for the decreased growth rates of the WAR mutants.

### Quantitative assessment of intracellular phage genomic DNA

**Figure 6:**
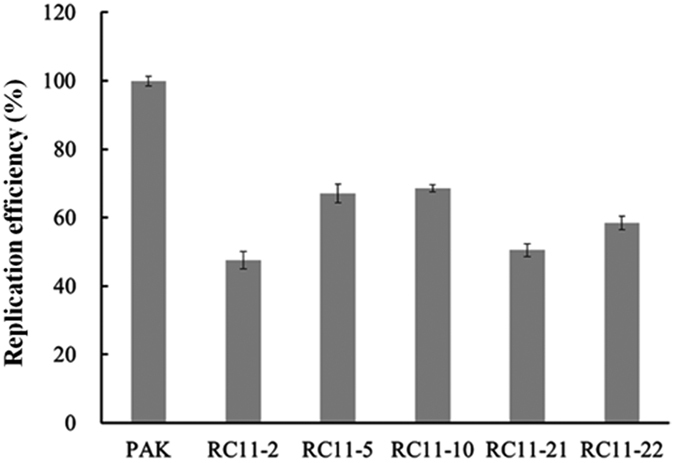
The quantitative analysis of the replication efficiency of the phage genomic DNA in strain PAK and the WAR mutants. The replication efficiency of the phage C11 genomic DNA in the WAR mutants was estimated by comparing with strain PAK after 30 min infection by phage C11.

To further probe if the phage genomic DNA was replicated in the WAR mutants, the cultures containing the WAR mutants and phage C11 were sampling at 0 min and 30 min for total DNA extraction, respectively. The same amount of DNA was used in the real-time quantitative PCR (RT-qPCR) analysis. At 0 min, all samples collected had the cycle threshold (Ct) value ranging from 24.10 to 24.49, indicating similar amounts of the phage particles were attached to the cellular surface of all strains. All samples collected at 30 min showed the Ct value ranging from 17.68 to 21.17, indicating the phage C11 genomic DNA was replicated intracellularly in the WAR mutants and strain PAK. Compared with the parent strain PAK, the five WAR mutants all had the genomic DNA replicated with different efficiencies, ranging from 47.6% to 68.6% (all P values less than 0.01) (Fig. 6). The results confirmed that the phage C11 genome had been successfully injected into bacterial cytoplasm and undergone DNA synthesis in the WAR mutants.

### Genome packaging analysis of the replicated DNA

**Figure 7:**
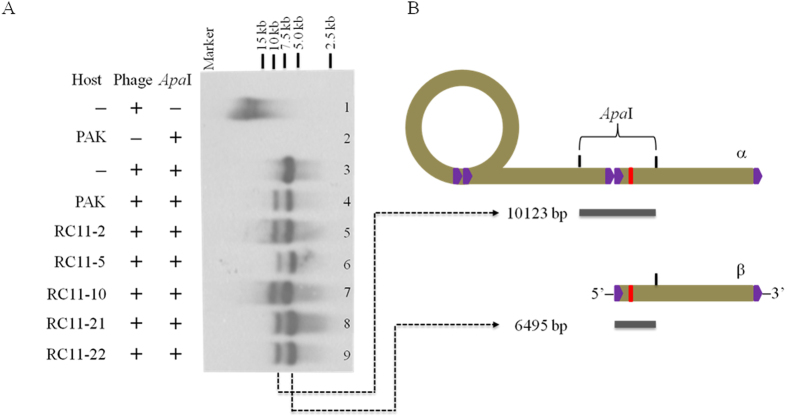
Analysis of the packaging process of the replicated genomic DNA by Southern blot. (**A**) total DNA was extracted from the samples (1–9), digested with the restriction enzyme *Apa*I, and detected by the synthesized DNA probe. The 10 kb *Apa*I fragment was predicted from the digested concatemeric DNA. The 6.5 kb *Apa*I fragment was predicted from the digested single viral genome (monomeric DNA). Marker: 15 kb DNA marker. (**B**) the schematic representation of the predicted phage genomic structures and the corresponding *Apa*I fragments detected in the Southern blot analysis. α: concatemeric DNA. β: monomeric DNA. The solid purple arrows stood for the terminal direct repeats (TDR) of the phage genome. The red rectangles stood for the DNA probe region.

The phage genomic DNA was synthesized in the WAR mutants without forming clear zones in the spotting assay or plaques in the double-layer plating analysis. The phage infection process might be blocked at the stages after genome replication. Southern blot analysis was performed to explore the packaging process of the synthesized phage genomic DNA. The data showed that the pure phage genomic DNA digested with *Xho*I, *Apa*I, *Eco*RV, and *Xba*I, respectively, displayed the positive bands with the expected sizes (Fig. S2). In the *Apa*I digested DNA samples prepared from the mixtures containing bacteria and phage C11, two positive bands were detected with the designed DNA probe, including the 10.0 kb fragment and the 6.5 kb fragment (Fig. 7A). The data was consistent with the predictions as shown in the scheme (Fig. 7B). The 10.0 kb fragment was possibly from the *Apa*I digested concatemeric DNA and the 6.5 kb fragment from the *Apa*I digested monomeric DNA (Fig. 7). All the data showed that the packaging process of the phage C11 genomic DNA may undergo normally in all the WAR mutants.

### Infection efficiency analysis in the WAR mutants

**Figure 8:**
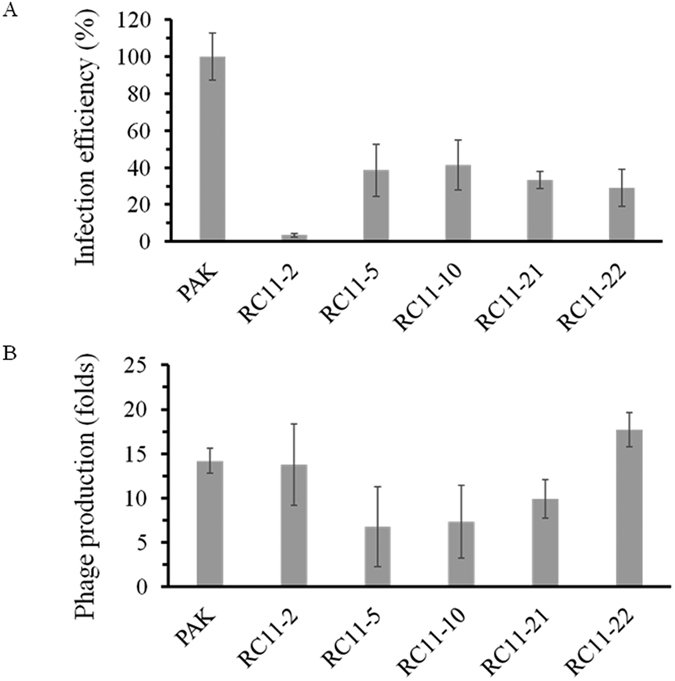
Infection efficiency assay of phage C11 infection in the WAR mutants. PAK instead of the WAR mutants was used as the indicator strain at a MOI of 0.00001 in the modified one-step growth experiment. (**A**) The infection efficiency was evaluated by comparing the phage titers of the WAR mutant cultures with that of the PAK cultures at 35 min. (**B**) The amount of phage progenies was evaluated by comparing the phage titer at 5 min with that at 35 min.

No transparent zone was observed in the lawn of the WAR mutants when infected with phage C11 in the spotting assay (Fig. 4B). The result was further confirmed by the fact that no plaques were detected in the standard double-layer plating method using the WAR mutants as indicators. However, turbid and small plaques were observed when plating the WAR mutant-phage complexes using strain PAK as the indicator, similar to the mixed-indicator technique^4^. The modified one-step growth experiment was performed to characterize the infectivity of phage C11 in the WAR mutants using wild type strain PAK as indicator instead of the WAR mutants. The WAR mutants RC11-2, RC11-5, RC11-10, RC11-21, and RC11-22 all could produce and release low quantities of phage C11, with the lower infection efficiency of 3.4%, 38.5%, 41.5%, 33.3%, and 29.0%, respectively (all P values less than 0.01) (Fig. 8A). The phage production ability was also estimated in the WAR mutants. The phage titres at 35 min were 13.8, 6.7, 7.3, 9.9, 17.7, and 14.2 folds to that at 5 min in RC11-2, RC11-5, RC11-10, RC11-21, RC11-22, and PAK, respectively, indicating phage progenies were obviously synthesized in the mutants (all P values less than 0.05) (Fig. 8B).

### Sensitivity analysis of the WAR mutants to phage K5 and K8

*P. aeruginosa* phage K5 and K8 were highly homologous to phage C11 at the genomic level and with respect of most individual genes with putative know functions, hence these viruses may share similar infection processes. However, with the spotting assay, the WAR mutants were found sensitive to the both phage K5 and K8, implying that the identified host genes were not required for the infection by phage K5 and K8. The data further showed the diversity of phage-host interactions.

## Discussion

*P. aeruginosa* phage C11 is highly similar to the genome of *P. aeruginosa* phage PaP1 and may be considered as a new member of the genus PaP1-like phages^36^ or PAK_P1-like phages^37^. Though the phages C11, JG004, PaP1, vB_PaeM_C2-10_Ab1, and PAK_P1 are genetically closely related to each other, they are the different phage isolates recovered across the world with their own evolutionary paths^38^. Phage C11 has 3 unique genes *ORF158, ORF159*, and *ORF165* in its genome. At the amino acid level, the hypothetical protein ORF158 has an identity of 41.12% with the pantothenate kinase encoded in *Prevotella falsenii*; ORF159 has an identity of 38.32% with the hypothetical protein encoded in *Pseudomonas veronii*; and ORF165 has an identity of 44.15% with the hypothetical protein PA13_1003640 encoding in *Pseudomonas aeruginosa* HB13. These genes may have their own bacterial origins and possibly be obtained by phage C11 via the process of horizontal gene transfer (HGT)^39^. The complete genome sequence of phage C11 is shown to be a liner DNA molecule with the 1173 bp identical terminal direct repeats, similar to a variety of *P. aeruginosa* phages which have highly conserved direct repeat sequences with length ranging from 184 bp to 1238 bp^37^,^40^.

A number of *Pseudomonas aeruginosa* phages recognize LPS as receptors for adsorption during infection, such as phage JG024^41^, JG004^32^, PaP1^36^, and KT28 and KTN6^31^. In our study, gene *wbpR* was found inactivated in the DAR mutant RC11-7. WbpR is a glycosyltransferase responsible for transferring the fourth residue L-Rhamnose of one O antigen repeating unit in the biosynthesis of OSA LPS^35^, indicating that LPS was the receptor for phage C11 binding. PA5181 is a putative molybdopterin oxidoreductase sharing a similarity of 76.11% with protein CbbB_c_ of *Ralstonia eutropha*^42^. For the first time, gene *PA5181* was confirmed related to the phage adsorption process via the unidentified pathway(s).

In the WAR mutants, five genes were unveiled essential for phage infection but not related to the steps of adsorption and injection. RT-qPCR and Southern blot analyses indicated that phage C11 had its genome replicated and packaged normally in the WAR mutants without detecting of phage progenies by using the standard spotting assay and the double-layer plating method. However, the modified one-step growth experiment method using PAK as indicator showed the WAR mutants can produce small quantities of phage C11, consistent with the data from the RT-qPCR and Southern blot analyses.

The functions of all the five disrupted genes weren’t investigated previously. BN889_05221 of *P. aeruginosa* PA38182 is a small hypothetical protein with unknown function. PA1115 of *P. aeruginosa* PAO1 is a hypothetical membrane protein probably with sulfuric ester hydrolase activity. PA1993 is a probable major facilitator superfamily (MFS) transporter sharing a homology of 50.2% with the L-arabinose efflux transporter Yhhs in *E. coli* MG1655^43^. Gene *PA0243* encodes a putative type III TetR regulator without knowing its function and target genes^44^,^45^. PA3808 has a similarity of 67.1% with IscX protein of ISC system FeS cluster assembly responsible for the ISC biogenesis in *E. coli*^46^,^47^. In the absence of protein IscX, the sulfur flux isn’t allowed to be distributed to the U34 thiolation process of tRNA^Glu^, tRNA^Gln^, and tRNA^Lys^ 8,48,49. The modification of the tRNAs plays an important role in determination of the ratio between protein gpG and gpGT of phage λ, and the ratio affects the efficiency of the assembly and release of phage λ progenies^48^. Iron ion can be one of the key components of the functional phage tail like protein complexes. In phage λ, the tail tip complex (TTC) mediates the adsorption and injection of the genomic DNA during the infection process. The TTC protein GpL has 4 conserved Cys residues at its C-terminal for the binding of the ISCs in which iron ion is obtained via the protein IscX^49^,^50^. In phage T4, seven iron ions were found coordinated by histidine residues arranged colinearly at the receptor-binding tip of the phage T4 long tail fiber with the crystal structure analysis^51^. In phage P2, the host-binding domain found in the tail-spike protein gpV of P2 phage also reveals a trimeric iron-binding structure with the crystal structure analysis^52^. Our data suggested the efficient production of phage C11 may require the biological availability of iron ion in the WAR mutant. More experiments are required to elucidate the underlying mechanisms.

There are three types of viral genome packaging systems, including energy-independent packaging system, portal-translocase system, and FtsK/HerA-type system^53^,^54^,^55^. In our work, the genome of phage C11 was identified to be a linear dsDNA molecule with 1173 bp TDR at its termini. Phages like λ and T7 package a precise genomic unit by cutting at the *cos* sites, while the other phages like SPP1 and P1 start packaging by cutting at the *pac* sites and finish packaging based on the size of the packaged DNA via the classical headful packaging mechanism^56^. With the discontinuous headful packaging model, phage T4 can package and transduce two plasmid DNAs with different antibiotic markers via the *in vitro* packaging experiments^57^. There’re packaging limits of genome lengths for phage production. For λ phage, the increasing genomic DNA length leads the dramatic reduction of phage infectivity^58^.

The head-to-tail concatemeric DNA and monomeric DNA were detected during the replication process with the Southern blot analysis in our work, similar to the genome replication and packaging process found in many tailed dsDNA phages. However, the *Eco*RI digested genomic DNA showed one unexpected 4.0 kb band, and the DNA samples purified from the phage infection process displayed the consistent results (Fig. S2A and B). With a series of experiments, the substitution of base A by base G was detected at the 1500 nt of the C11 genome with the sequence analysis, generating a new *Eco*RI site in part of the phage C11 population (Fig. S2C). The data may imply relatively high mutation rates in the genome of phage C11^59^,^60^.

*P. aeruginosa* phage K5 and K8 are highly homologous to phage C11 with the overall similarities of 94.81% and 94.82%, respectively. Most genes with known putative functions also share high similarities among the three genomes. Additionally, the three phages recognize OSA LPS as the receptors^61^,^62^. However, the WAR mutants were sensitive to both phage K5 and K8 in the spotting assay, implying that the two phages may harness other infection pathways for successful phage infection. Probably the differences of the hypothetical proteins help phage K5 and K8 overcome the host barrier and produce phage progenies more efficiently than phage C11. The result further indicates the evolved diversity and complexity of the phage-host interactions from both bacteria and virus. More studies are needed to unveil the mechanisms underlying the arm race between host and phage.

## Materials and Methods

### Bacterial strains, phage, plasmids, and growth conditions

The bacterial strains and the plasmids used in this study were listed in Table 2. Phage C11 was a virulent virus isolated from fresh water river samples using the clinical strain *P. aeruginosa* TJC422 as indicator^33^. LB medium was used for routine incubations of bacteria. If necessary, antibiotics with appropriate concentrations were added to the cultures, carbenicillin (150 μg/ml) and gentamicin (100 μg/ml) for *P. aeruginosa* incubations; ampicillin (100 μg/ml) and gentamicin (10 μg/ml) for *E. coli* incubations. All incubations were performed at 37 °C.

### Characterization of phage C11

Phage particles of C11 for transmission electron microscopy (TEM) were prepared and purified as described previously^63^. Briefly, the purified phage C11 solution was added on the carbon film over the copper grid for negative staining with 2% phosphotungstic acid (pH 6.7). Philips EM400ST transmission electron microscope was used for phage visualization. Purified phage particles were also subjected to SDS-PAGE for the viral structural proteins analysis. *P. aeruginosa* PAK was used as the indicator strain to determine the latent period and the burst size of phage C11 by one-step growth experiment as described previously at a MOI of 0.001. Triplicate cultures were involved in the experiment. Samples were diluted adequately and phage titers were measured by the double-layer plate method^64^.

### Phage genome sequencing and identification of the terminal sequences

The phage genomic DNA was extracted and purified for genome sequencing^65^. The whole genome sequencing was conducted by using the Hiseq Illumina 2500 System in GENEWIZ, Inc., China (http://www.genewiz.com/). The obtained sequences were filtered with the software Trimmomatic (v0.30) to remove the sequences with low quality and the adaptor sequences^66^. The total clean data included 6,983,460 reads with 692,093,907 bp and was assembled into a single contig with the software Velvet_v1.2.10^67^.

Termini identification was conducted according to the procedure described previously^61^. The HFS of the assembled genome which might represent the phage genome termini of were revealed by analyzing the sequencing depth across the genome^34^. The software DNAMAN was used to simulate the restriction mapping of the C11 genome on the basis of the termini inferred from the HFS sites. The *Pvu*I and *Afl*II digested DNA fragments that possibly contained the termini of phage C11 were recovered from the electrophoresis gels, filled in with the large fragment (klenow), and ligated with T4 DNA ligase. The ligation products were amplified with the primers listed in Table 3 3F and 3R for 3′ terminus determination and 5 F and 5 R for 5′ terminus determination. The amplicons were sequenced to determine the terminal sequences. The assembled genome sequence was curated and deposited in GenBank of NCBI with the accession number KT804923.

### Genome annotation and comparative genomic analysis

The software tRNAscan-SE v1.21 was performed to predict the tRNA encoding genes^68^. Possible protein coding genes were predicted with DNA Master 5.0.2 (http://cobamide2.bio.pitt.edu/) and the threshold value was set at 100 bp. The annotation was carried out by searching against the non-redundant protein database (nr) from NCBI using the alignment software blast-2.2.27+ 69 and the tool provided at the website http://stothard.afns.ualberta.ca/cgview_server/70. The genome sequences of phage PaP1 (NC_019913), PAK_P1 (NC_015294), JG004 (NC_019450), vB_PaeM_C2-10_Ab1 (NC_019918), K5 (KU497559), and K8 (KT736033) in FASTA format were downloaded from the NCBI genome database for comparison analysis.

### Isolation and characterization of phage resistant mutants

Tn5G transposon insertional library was constructed as described previously^71^. *E. coli* DH5α/pRK2013Tn5G and *P. aeruginosa* PAK were used as the donor strain and the recipient strain, respectively. The library was incubated with 5 ml phage C11 stock solution (10^11^ pfu/ml) for 4 h to enrich phage-resistant mutants. Phage-resistant mutants were screened on LB plates with gentamicin (100 μg/ml) and ampicillin (100 μg/ml). The adsorption rate of the isolated phage-resistant mutants was analyzed as previously described. Briefly, MOI of 0.001 was used in the test. After adsorption for 10 min, free phage particles were removed by centrifugation for titer analysis by the double-layer plate method^64^. To verify if the DAR mutants can resume their adsorption rates as strain PAK in the extended period, the time-course of adsorption rate was analyzed at a MOI of 0.0001. The cultures were sampled at 2 min intervals for further analysis of the free phage particles.

### Identification of the disrupted genes by inverse PCR

To identify the insertional site of transposon Tn5G in the phage resistant mutants, inverse PCR method was performed as described^72^. Briefly, bacterial genomic DNA was digested completely by the restriction endonuclease *Taq*I. The digested DNA fragments were subsequently subjected to self-ligation. The ligated molecules were amplified using the primer pair OTn1 and OTn2 listed in Table 3. PCR products were sequenced and the obtained sequences were analyzed by searching the database GenBank of NCBI (http://www.ncbi.nlm.nih.gov/) and the *Pseudomonas* genome database (http://www.pseudomonas.com/).

### Complementation assay

The genomic structure of the disrupted gene was analyzed by searching the website (http://www.pseudomonas.com/) and the promoter region of the genes were predicted in the website (http://fruitfly.org/seq_tools/promoter.html). The target genes for complementation tests were amplified by PCR using the primers listed in Table 3. The shuttle vector pUCP18 was used for the target gene cloning. Some genes were driven by their natural promoters and other genes located with operons were driven by the *Plac* promoter on the vector. The recombinant plasmids were transformed into the corresponding mutants by electroporation. The sensitivity of transformants to phage C11 was analyzed by the spotting assay in which 10^5^–10^6^ phage particles were applied in each spot.

### Growth dynamic assay of the phage-resistant mutants

The growth rate of the phage-resistant mutants was analyzed by incubation in 96-well plates in quadruplicate. At the beginning of incubation, phage C11 was added to the cultures at various MOIs (multiplicity of infection). The optical density of the cultures was measured at the wavelength of 600 nm.

### Real-time quantitative PCR analysis of phage genomic DNA

The phage resistant mutants were infected with phage C11 according to the procedure previously used for one-step growth experiment at a MOI of 0.0001^64^. The samples were collected at 0 min and 30 min, respectively. Total DNA including phage genomic DNA was extracted as previously described^65^. The same amount of the purified DNA of each samples was used as templates for real-time quantitative PCR to measure the amount change of phage genomic DNA. Go Taq® qPCR Master Mix from Promega was used. Gene (*ORF056*) encoding RNA polymerase in phage C11 genome was chosen as the amplified target and the primers were designed (Table 3). Bacterial genomic DNA of strain PAK and water were used as negative controls in the analysis. The experiment was repeated at 3 times and each sample was analyzed in triplicate. The Ct (cycle threshold) value was used to evaluate the amount of the target templates.

### Southern blot analysis

Bacterial culture of 40 ml was incubated to logarithmic phase (OD_600_ = 0.6). Harvested bacterial cells were resuspended in fresh LB medium and mixed with phage C11 at a MOI of 1. After 1 min adsorption, free phage particles in supernatant were removed by centrifugation, and cell pellet was transferred into 100 ml LB medium for incubation. Samples were removed at 30 min for total DNA preparation. The purified DNA was digested with the restriction enzyme *Apa*I, *Eco*RI, *Eco*RV, *Xho*I, and *Xba*I, respectively, and subjected to Southern blot analysis as described previously^65^. The primers S1 and S2 were used to synthesize the probe of 520 bp for detecting the genomic DNA of phage C11 (Table 3 and Fig. S2A). The probe was labelled by using DIG High Prime DNA labeling and Detection Starter Kit II (Roche).

One unexpected 4.0 kb *Eco*RI band was detected in the Southern blot analysis. It’s predicted that one new *Eco*RI site was generated by spontaneous mutation in the C11 genome. To verify this hypothesis, the primers SM-F and S2 were used to amplify the 5′ terminal fragment and it was subsequently digested with *Eco*RI enzyme (Table 3 and Fig. S2). The 1.3 kb *Eco*RI fragment was purified from gel and sequenced directly to verify the existence of the new *Eco*RI site.

### Infection efficiency analysis in the WAR mutants

The standard one-step growth experiment was performed as described previously with modifications^64^. In brief, the same amount of phages and the same amount of host cells were used in all cultures at a MOI of 0.00001 in the test. After 1 min adsorption, free phage particles were removed by centrifugation and the pellet was resuspended in fresh LB medium. Repeat the step for three more times and the cell-phage complexes were finally resuspended in 100 ml medium. PAK strain was used as the indicator instead of the WAR mutants in the double-layer plating method to count the infection centres. The plaques were counted at 5 min and 35 min, respectively. The infection efficiency at 35 min was evaluated by comparing the phage titers of the WAR mutant cultures with that of the PAK cultures. The phage production of phage C11 was evaluated by comparing the titers at 5 min with that at 35 min. Triplicate cultures were involved in the experiment.

### Sensitivity testing with *Pseudomonas aeruginosa* phage K5 and K8

The sensitivity of the isolated phage-resistant mutants to *P. aeruginosa* phage K5 and K8 were performed with the spotting assay method as described previously^64^.

## Additional Information

**How to cite this article**: Cui, X. *et al*. Characterization of *Pseudomonas aeruginosa* Phage C11 and Identification of Host Genes Required for Virion Maturation. *Sci. Rep.* **6**, 39130; doi: 10.1038/srep39130 (2016).

**Publisher's note:** Springer Nature remains neutral with regard to jurisdictional claims in published maps and institutional affiliations.

## Supplementary Material

Supplementary Information

## Figures and Tables

**Table 1:** Phage genes with putative functions.

**Category**	**ORF**	**Function**
Nucleotide metabolism	ORF020	putative nicotinamide phosphoribosyl transferase
	ORF022	phosphoribosyl pyrophosphate synthetase
	ORF023	putative ATPase
	ORF024	putative RNA ligase
	ORF028	putative phosphoesterase
	ORF030	putative phosphohydrolase
	ORF033	DNA ligase
	ORF034	putative dCMP deaminase
	ORF044	putative protease subunit
Head and tail assembly	ORF049	putative large terminase subunit
	ORF050	structural protein
	ORF051	N6 adenine specific DNA methyltransferase
	ORF054	major capsid protein
	ORF056	putative RNA polymerase
	ORF059	putative structural protein
	ORF065	putative tape measure protein
	ORF069	putative baseplate protein
	ORF071	putative base plate related protein
	ORF073	putative tail fiber protein
	ORF075	putative tail fiber protein
DNA replication, transcription, recombination and modification	ORF086	putative RNA ligase
	ORF101	DNA primase/helicase
	ORF102	DNA polymerase
	ORF109	putative exodeoxyribonuclease
	ORF123	thymidylate synthase
	ORF125	ribonucleoside diphosphate reductase beta subunit
	ORF126	ribonucleoside diphosphate reductase alpha subunit
Cell lysis	ORF031	putative cell wall hydrolase
	ORF076	endolysin

**Table 2:** Strains, phage, and plasmids used in this study.

Strain, phage, and plasmid	Description	Reference
*P. aeruginosa*		
PAK	Laboratory strain	73
RC11-2	*BN889_05221*::Tn5G, phage C11 resistant mutant of PAK, Gm^r^	This study
RC11-5	*PA3808*::Tn5G, phage C11 resistant mutant of PAK, Gm^r^	This study
RC11-7	*wbpR*::Tn5G, phage C11 resistant mutant of PAK, Gm^r^	This study
RC11-10	*PA1993*::Tn5G, phage C11 resistant mutant of PAK, Gm^r^	This study
RC11-20	*PA5181*::Tn5G, phage C11 resistant mutant of PAK, Gm^r^	This study
RC11-21	*PA0243*::Tn5G, phage C11 resistant mutant of PAK, Gm^r^	This study
RC11-22	*PA1115*::Tn5G, phage C11 resistant mutant of PAK, Gm^r^	This study
Phage		
C11	*P. aeruginosa* lytic phage	33
Plasmid		
pUCP18	Broad host range shuttle vector, Ap^r^	74
pRK2013Tn5G	used for transposon mutagenesis, Gm^r^	71
pXL1502	*BN889_05221* gene driven by *P*_*lac*_ promoter cloned in pUCP18, Ap^r^	This study
pXL1503	*PA3808* gene driven by *P*_*lac*_ promoter cloned in pUCP18, Ap^r^	This study
pXL1504	*wbpR* gene with promoter region cloned in pUCP18, Ap^r^	This study
pXL1505	*PA1993* gene with promoter region cloned in pUCP18, Ap^r^	This study
pXL1506	*PA5181* gene driven by *P*_*lac*_ promoter cloned in pUCP18, Ap^r^	This study
pXL1507	*PA0243* gene driven by *P*_*lac*_ promoter cloned in pUCP18, Ap^r^	This study
pXL1508	*PA1115* gene with promoter region cloned in pUCP18, Ap^r^	This study

^*^Ap^r^, ampicillin resistance; Gm^r^, gentamicin resistance.

**Table 3:** Primers used in the study.

Primer	*Primer sequence (5′-3′)	Target region
OTn1	GATCCTGGAAAACGGGAAAG	genome DNA regions flanking by Tn5G
OTn2	CCATCTCATCAGAGGGTAGT	
BN889_05221-F	CCCAAGCTTGAACGGACGCTCTTTGTG	coding region of gene *BN889_05221*
BN889_05221-R	CGCGGATCCACACCTTGCGTGAACTGG	
PA3808-F	CGCGGATCCGCCAAACAGTTCATCACCACC	coding region of gene *PA3808*
PA3808-R	CGCGGATCCTTTTCCCAATGCCCGCCCTTCC	
wbpR-F	CGCGGATCCCAACAAGCCGCTGAAGCC	gene *wbpR* with its natural promoter
wbpR-R	CGCGGATCCAGAACACCGACGCCCTGG	
MFS-F	CGCGGATCCGGCACAACCGATTAGACG	gene *PA1993* with its natural promoter
MFS-R	CGCGGATCCTACCGACCAGACTCAGGGA	
PA5181-F	CGCGGATCCGACAACCTCAACCAGCACT	coding region of gene *PA5181*
PA5181-R	CGCGGATCCGCCGAGCCCTATTCCTT	
PA0243-F	CCCAAGCTTGCGGTGATGCGGTTGGTG	coding region of gene *PA0243*
PA0243-R	CCCAAGCTTGCGGGAGAGGCAGTCGGT	
PA1115-F	CCCAAGCTTGAACGGACGCTCTTTGTG	gene *PA1115* with its own promoter
PA1115-R	CGCGGATCCACACCTTGCGTGAACTGG	
56-F	GTTGGTTTCTTCCCCGAGG	qPCR primers, coding region of gene *ORF056*
56-R	ATTCGTCTGCCTTCCATCGC	
3 F	TATCAGATGGAGGATGTTCAC	3′ terminus of phage C11 genome
3 R	GCTGAGAGAGACCTTGTTCC	
5 F	GGTGTTCCATCCACTCCCTG	5′ terminus of phage C11 genome
5 R	GTTCGCCTTCTGCCAGTTAT	
S1	AGCCTGCTCTCTCCGTTC	The DNA probe for Southern blot analysis
S2	TCTCAAGAGTGCTGTCCC	
SM-F	ACACTGAAAACCTTGACTCC	Amplification of the fragment with a mutation generating a *Eco*RI site
S2	TCTCAAGAGTGCTGTCCC	

^*^The bases underlined represent the added restriction enzyme sites.
