# Supplementary material for: Characterization of Pseudomonas aeruginosa Phage C11 and Identification of Host Genes Required for Virion Maturation
Source: Sci Rep. 2016 Dec 21;6:39130. doi: 10.1038/srep39130 (PMC5175280; doi:10.1038/srep39130)
Supplement: Supplementary Information [file srep39130-s1.pdf]

Characterization of *Pseudomonas aeruginosa* Phage C11 and Identification of Host Genes Required for Virion Maturation

Xiaoli Cui, Jiajia You, Li Sun, Xiaojing Yang, Tian Zhang, Kechong Huang, Xuewei Pan, Fenjiao Zhang, Yang He, Hongjiang Yang*

* corresponding author:
hongjiangyang@tust.edu.cn

Fig. S1

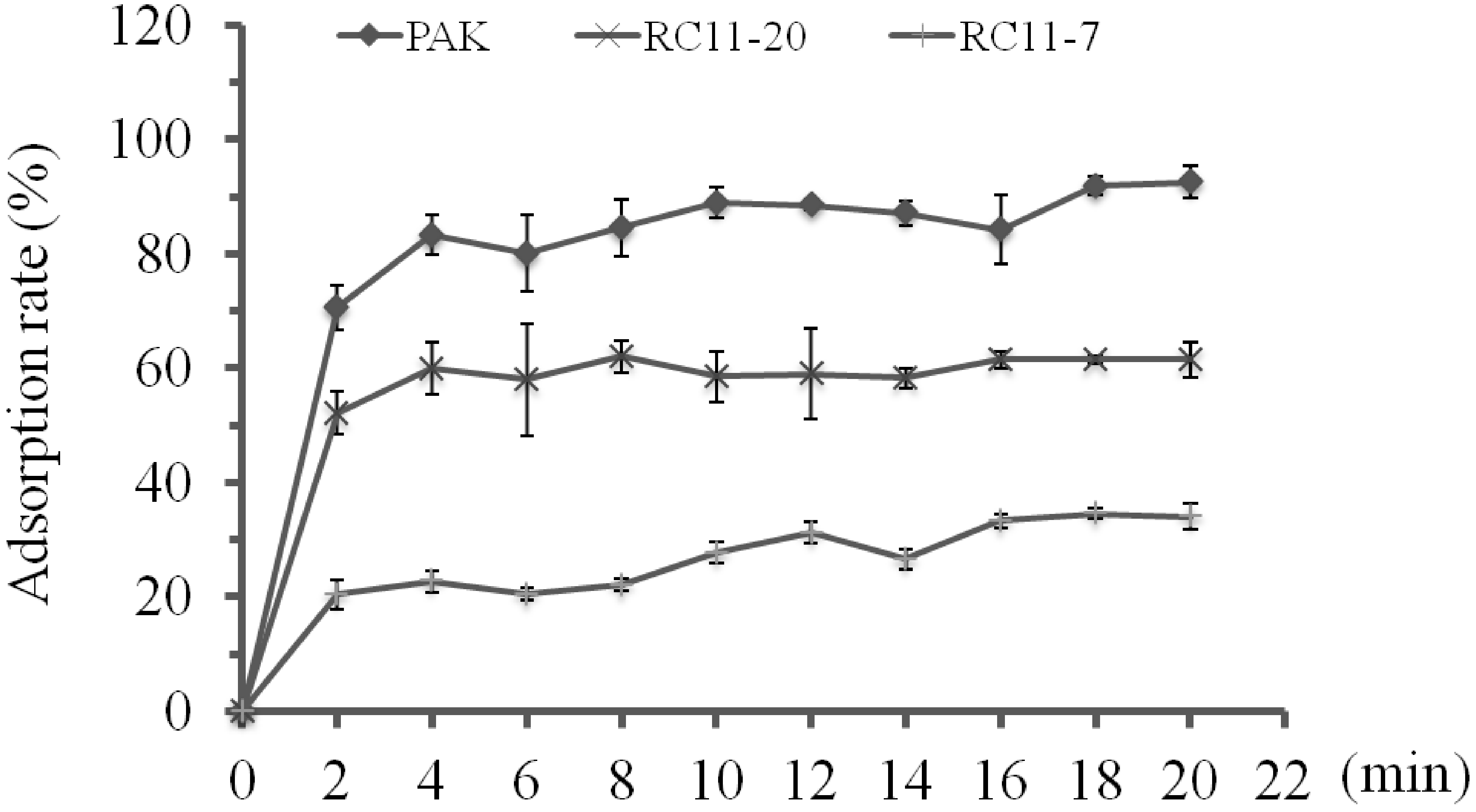

Fig. S1. Time-course analysis of the adsorption rates in the DAR mutants. MOI of 0.0001 was used in the experiments. The experiment involved triplicate cultures. PAK was used as control and the indicator strain.

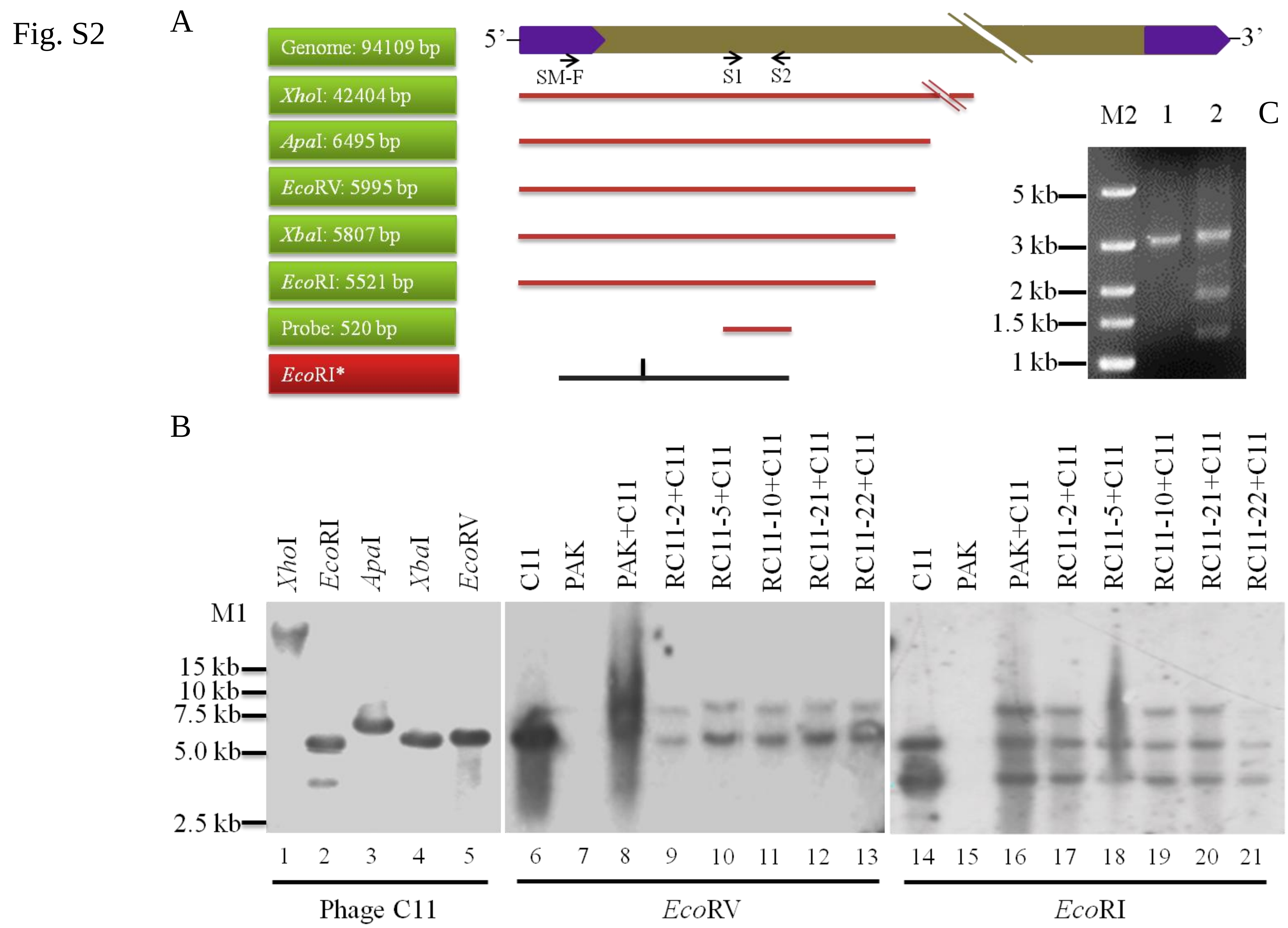
Fig. S2
A
Genome: 94109 bp
XhoI: 42404 bp
ApaI: 6495 bp
EcoRV: 5995 bp
XbaI: 5807 bp
EcoRI: 5521 bp
Probe: 520 bp
EcoRI*
5'
3'
SM-F
S1
S2
C
M2
1
2
5 kb
3 kb
2 kb
1.5 kb
1 kb
B
M1
15 kb
10 kb
7.5 kb
5.0 kb
2.5 kb
XhoI
EcoRI
ApaI
XbaI
EcoRV
C11
PAK
PAK+C11
RC11-2+C11
RC11-5+C11
RC11-10+C11
RC11-21+C11
RC11-22+C11
1 2 3 4 5 6 7 8 9 10 11 12 13 14 15 16 17 18 19 20 21
Phage C11
EcoRV
EcoRI

Fig. S2. Analysis of the phage genomic DNA with the Southern blot method. A: The scheme of the expected size of the positive bands detected by the DNA probe. Words in green boxes showed the genome size, the DNA probe size, and the fragment sizes generated from the restriction enzyme digestions, including *Xho*I, *Apa*I, *Eco*RV, *Xba*I, and *Eco*RI. Words in the red box indicated the new *Eco*RI site generated from the spontaneous mutation in the C11 genome. The primers S1 and S2 were used to synthesize the DNA probe. The primers SM-F and S2 were used to amplify the fragment that may including a new *Eco*RI site. B: The analysis of various DNA samples (1-21) digested with various restriction enzymes as indicated, including *Xho*I, *Apa*I, *Eco*RV, *Xba*I, and *Eco*RI. The samples treated with *Eco*RI enzyme showed the unexpected positive band with the size of 4.0 kb. C: Electrophoresis of the fragment possibly containing one new *Eco*RI site with the digestion of *Eco*RI enzyme. A portion of the PCR product was cut by the enzyme *Eco*RI and the result was confirmed by sequence analysis. M2 stood for 5 kb DNA marker. 1 stood for the PCR product. 2 stood for the PCR product digested with the *Eco*RI enzyme.
